# Quantitative Evaluation of Oxygen Extraction Fraction Changes in the Monkey Brain during Acute Stroke by Using Quantitative Susceptibility Mapping

**DOI:** 10.3390/life13041008

**Published:** 2023-04-13

**Authors:** Yuguang Meng, Chun-Xia Li, Xiaodong Zhang

**Affiliations:** 1 EPC Imaging Center, Emory National Primate Research Center, Emory University, Atlanta, GA 30329, USA; 2 Division of Neuropharmacology and Neurologic Diseases, Emory National Primate Research Center, Emory University, Atlanta, GA 30329, USA

**Keywords:** MCAO, QSM, OEF, nonhuman primate

## Abstract

Background: The oxygen extraction fraction (OEF) indicates the brain’s oxygen consumption and can be estimated by using the quantitative susceptibility mapping (QSM) MRI technique. Recent studies have suggested that OEF alteration following stroke is associated with the viability of at-risk tissue. In the present study, the temporal evolution of OEF in the monkey brain during acute stroke was investigated using QSM. Methods: Ischemic stroke was induced in adult rhesus monkeys (n = 8) with permanent middle cerebral artery occlusion (pMCAO) by using an interventional approach. Diffusion-, T2-, and T2*-weighted images were conducted on day 0, day 2, and day 4 post-stroke using a clinical 3T scanner. Progressive changes in magnetic susceptibility and OEF, along with their correlations with the transverse relaxation rates and diffusion indices, were examined. Results: The magnetic susceptibility and OEF in injured gray matter of the brain significantly increased during the hyperacute phase, and then decreased significantly on day 2 and day 4. Moreover, the temporal changes of OEF in gray matter were moderately correlated with mean diffusivity (MD) (r = 0.52; *p* = 0.046) from day 0 to day 4. Magnetic susceptibility in white matter progressively increased (from negative values to near zero) during acute stroke, and significant increases were seen on day 2 (*p* = 0.08) and day 4 (*p* = 0.003) when white matter was significantly degenerated. However, significant reduction of OEF in white matter was not seen until day 4 post-stroke. Conclusion: The preliminary results demonstrate that QSM-derived OEF is a robust approach to examine the progressive changes of gray matter in the ischemic brain from the hyperacute phase to the subacute phase of stroke. The changes of OEF in gray matter were more prominent than those in white matter following stroke insult. The findings suggest that QSM-derived OEF may provide complementary information for understanding the neuropathology of the brain tissue following stroke and predicting stroke outcomes.

## 1. Introduction

Quantitative susceptibility mapping (QSM) is a novel and non-invasive MRI technique that can characterize the spatial distribution of magnetic susceptibility within the brain tissue by solving the field-to-source problem in data post-processing of T2*-weighted MR images [1,2,3]. Recent studies have demonstrated QSM’s applications in assessing cerebral iron deposition, demyelination, and quantification of venous oxygen saturation in the brain with neurodegenerative diseases [4]. Susceptibility-weighted imaging (SWI) has been used to assess the abnormal magnetic susceptibility of the brain (e.g., for hemorrhage due to stroke or traumatic injury) [5,6]. QSM has recently attracted greater interest in stroke research, as it might be able to provide unique information to assess the tissue viability in ischemic hypoperfusion areas of the brain [7,8,9,10,11], as compared to diffusion and perfusion MRI, which is often used to examine the brain tissues at risk in clinical practice or research of animal models [12,13,14,15,16,17]. It has also been suggested that the increase and decrease in magnetic susceptibility in the stroke brain might be related to the tissue viability status after stroke, as reported in a recent study of a canine model of stroke [8]. 

The oxygen extraction fraction (OEF) is the ratio of blood oxygen that the brain tissue takes from the blood flow, reflecting the efficiency of oxygen utilization by the tissue. OEF can be directly measured using the ^15^O_2_ positron emission tomography (PET) technique (PET-OEF). Recent studies of patients suggest that OEF can be estimated by examining the susceptibility differences between the cortical veins and surrounding tissues using the QSM approach (QSM-OEF) [18], and a good correlation was seen between PET-OEF and QSM-OEF, as demonstrated in previous studies of stroke patients [9,19]. The prior results suggest that QSM-OEF may be an effective approximation to estimate OEF in the brain non-invasively. 

As OEF is closely related to the tissue’s functionality, it has been used to evaluate the tissue viability in ischemic brains in recent QSM studies of patients [20,21,22], as well as in a PET imaging study of stroke baboons [23]. Diffusion-weighted imaging (DWI) is highly sensitive to detect ischemic infarction, and DWI and OEF mismatch (DWI-OEF) was suggested to be more specific to define the ischemic penumbra compared to traditional DWI and perfusion-weighted image (DWI-PWI) mismatch, as reported in a rat study of stroke [24]. A recent study of patients suggested that the tissue volumes with increased QSM-OEF values could be used to predict the penumbra volumes of patients who were admitted <24 h after stroke onset, showing the potential of QSM-OEF to be used as a penumbra biomarker to guide treatment selection in acute stroke patients [25]. The spatiotemporal evolution of QSM and OEF in ischemic brains has been explored in a few studies of stroke patients but remains poorly understood due to substantial variation in the stroke onset times of admitted patients and the very limited timepoints of data collection during acute stroke. In the present study, we aimed to examine the longitudinal evolution of oxygen extraction fraction (OEF) in the ischemic brain using a rhesus monkey model of stroke. 

## 2. Methods and Materials

Adult healthy rhesus monkeys (n = 8, female, 10–21 years old, 8.0 ± 1.5 kg) were utilized. The stroke surgery was performed using a minimally invasive interventional approach in which a microcatheter was navigated and guided with a parent catheter to a distal cerebral artery of the middle cerebral artery (MCA) through the femoral artery to induce permanent occlusion (pMCAO) in the right hemisphere of the brain, as described previously [26]. The MRI data were collected on day 0 (3.5–6 h post stroke) (n = 8), day 2 (48 h, n = 6), and day 4 (96 h, n = 4). The MRI data were not acquired more frequently due to concerns about the effects of prolonged anesthesia administration on the day of surgery (day 0) and repeated anesthesia on cognitive dysfunction and postoperative recovery [27]. During the MRI scans, the stroke monkeys were anesthetized with 1.0–1.5% isoflurane and immobilized in a supine position using a custom-made head holder. Vital parameters, including Et-CO_2_, inhaled CO_2_, O_2_ saturation, blood pressure, heart rate, respiration rate, and core body temperature, were monitored continuously and regulated, and body temperature was maintained at 37 °C. The QSM scan of one stroke monkey was interrupted on day 0 but resumed on day 2 and day 4 post-stroke. The animals were euthanatized immediately following their last MRI scans without recovery from anesthesia. The monkey brains were harvested and fixed for hematoxylin and eosin (H&E) and fast blue staining for validation of stroke lesions. All procedures for animal care, surgery, and MRI scans were approved and were in compliance with the Institutional Animal Care and Use Committees (IACUC) of the institute and the National Institutes of Health (NIH) guidelines for the care and use of laboratory animals.

### 2.1. MRI Data Acquisition

The rhesus monkeys were scanned with a Siemens 3T TIM Trio scanner (Siemens Healthineers, Erlangen, Germany) using a Siemens 8-channel high-resolution phased-array volume knee coil. T2*-weighted images were acquired with a multi-echo 2D spoiled gradient-recalled echo (GRE) sequence with the following imaging parameters: echo time (TE) = 5 ms–80 ms, 5 echoes, flip angle = 70°, time of repetition (TR) = 1400 ms, data matrix size = 128 × 128, slice thickness 1.5 mm, in-plane resolution = 0.75 mm × 0.75 mm. T2-weighted images were acquired with a multi-slice spin-echo sequence with TE = 8–146 ms, 4 echoes, TR = 5.8 s, matrix size = 128 × 128, slice thickness = 1.5 mm, and in-plane resolution = 0.75 mm × 0.75 mm. The diffusion-weighted images were collected with an echo planar imaging (EPI) pulse sequence with the Generalized Autocalibrating Partially Parallel Acquisition technique (GRAPPA, R = 3) and the following parameters: TE/TR = 80 ms/5 s, data matrix = 64 × 64, in-plane resolution = 1.5 mm × 1.5 mm, slice thickness = 1.5 mm, and 30 gradient directions with b-value = 0, 1000 s/cm^2^. For the field inhomogeneity correction, a field map for the whole brain was acquired with a standard gradient echo sequence with TE = 6.2 ms and 8.7 ms, TR = 500 ms, slice thickness = 1.3 mm, FOV = 96 mm × 96 mm, and in-plane resolution = 1.3 mm × 1.3 mm. T1-weighted images (T_1w_) were acquired by using a 3-dimensional (3D) magnetization-prepared rapid acquisition gradient echo (MPRAGE) sequence with GRAPPA (R = 2), inversion time = 950 ms, TE/TR = 3.5 ms/3 s, and isotropic resolution = 0.5 mm. The high-resolution susceptibility-weighted imaging (SWI) was acquired using a 3D gradient-recalled echo pulse sequence for examination of hemorrhagic transformation of the brain following stroke.

### 2.2. MRI Data Processing

Quantitative susceptibility maps were reconstructed from the acquired multi-echo GRE image data by using the morphology-enabled dipole inversion (MEDI) method [1]. The transverse relaxation rates, R_2_ (1/T2) and R_2_* (1/T2*) were calculated with corresponding T2- or T2*-weighted images with different echo times (TEs) and conventional exponential fits with custom-written MATLAB scripts (MathWorks, Inc). By using the FSL toolbox (FMRIB; Oxford, UK), image distortion correction was performed on each of the DWI data with the acquired field map. Furthermore, the DTI indices (fractional anisotropy (FA) and mean diffusivity (MD)) were calculated by using the diffusion toolbox in the FSL software.

**Figure 1:**
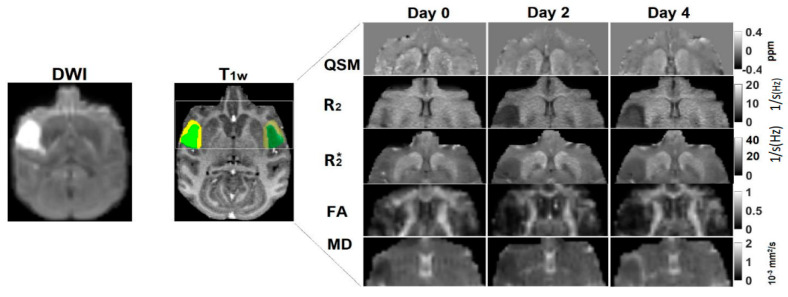
Demonstration of ischemic lesion in a stroke monkey brain (RJJ3): (**Left**) Diffusion-weighted image (DWI) of the stroke brain (day 4). (**Middle**) The infarcted area defined by DWI on day 4 was segmented into the white matter region (WM; non-transparent yellow color) and gray matter region (GM; non-transparent green color) on the baseline T1-weighted image (T_1_w) of the same monkey. The homologous region (transparent color) was illustrated on the contralateral side of the T1-weighted image. (**Right**) The corresponding maps of quantitative susceptibility, R_2_, R_2_*, FA, and MD of the same monkey brain on day 0, day 2, and day 4. QSM, quantitative susceptibility map; R_2_, R_2_*, transverse relaxation rate; FA, fractional anisotropy; MD, mean diffusivity.

As shown in Figure 1, the MCAO lesion regions of one stroke monkey were illustrated on the last diffusion-weighted images (DWI) acquired at each time point. The lesion regions were determined from the DWI voxels, where the image intensity was above threefold that of the average for the whole brain. The gray matter and white matter in the ischemic areas of each monkey were derived from the stroke lesion region defined by the last DWI scan of the same monkey and extracted at each timepoint by using the segmentation on the baseline T_1_w images of the monkey scanned before the stroke surgery. The maps of QSM, R_2_, R_2_*, FA, and MD of each monkey were registered (with 12 DOF linear affine transformation) to the baseline T_1_w images of the same monkey. R_2_’ is defined as the difference between R_2_ and R_2_* (R_2_’ = R_2_*–R_2_) and was calculated because it is associated with OEF, as reported in a previous report of stroke patients [28]. The values of each MRI parameter in the segmented gray matter and white matter of lesion areas and the corresponding regions on the contralateral side of the brain were extracted and averaged. 

The veins were detected based on the QSM threshold method, where the veins were determined by using a 16 × 16 pixel convolution kernel to extract the pixels with brightness greater than the mean + 2× standard deviation in the kernel, as reported previously [19,29]. The oxygen extraction fraction was calculated by using the formula OEF = Δχ × *pv*/(Δχ_do_ × *Hct*), where Δχ is the susceptibility difference between the veins and the surrounding brain tissues, *Pv* (=7.0) is a partial volume effect correction factor, Δχ_do_ (=0.18 ppm [cgs]) is the susceptibility difference per unit of hematocrit between fully deoxygenated vs. oxygenated blood, and *Hct* (0.45) is hematocrit [19].

Statistical analysis was conducted using the SPSS 17.0 software (SPSS Inc., Chicago, IL, USA). In order to determine the differences between hemispheres or the post-occlusion timepoints for QSM, R_2_, OEF, FA, and MD in the white matter and gray matter of infarction areas, a two-way multivariate analysis of variance (MANOVA) was carried out with the independent factors hemisphere (contralateral vs. ipsilateral) and the post-occlusion timepoints (days 0, 2, and 4), followed by post hoc analysis (with *p* < 0.05 as the significance threshold) and Bonferroni correction. The interaction effects between hemispheres and the post-occlusion timepoints were also examined. Pearson’s correlation analyses were performed between the QSM-derived OEF and DTI-derived parameters in the gray matter or white matter lesion regions of the ipsilateral hemisphere of the brain.

## 3. Results

**Figure 2:**
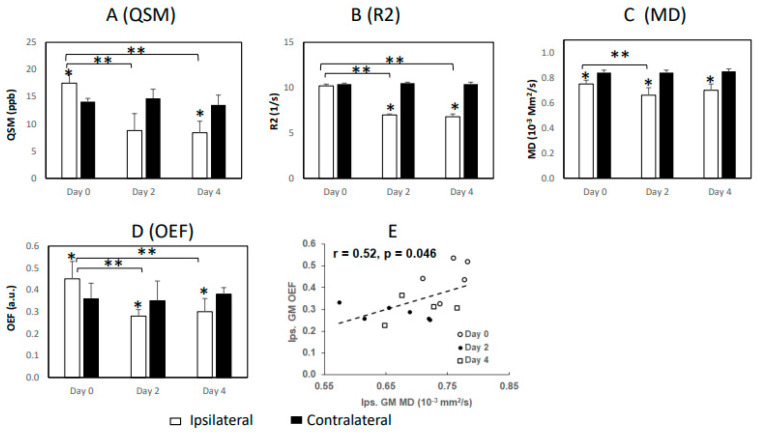
Demonstration of longitudinal changes in (**A**) magnetic susceptibility (QSM), (**B**) transverse relaxation rate (R_2_), (**C**) mean diffusivity (MD), (**D**) oxygenation extraction fraction (OEF), and (**E**) temporal correlation (OEF vs. MD) in the gray matter (GM) of ischemic monkey brains during acute stroke; * *p* < 0.05 compared to the contralateral side; ** *p* < 0.05 compared to the days post-occlusion; ppb: parts per billion.

**Figure 3:**
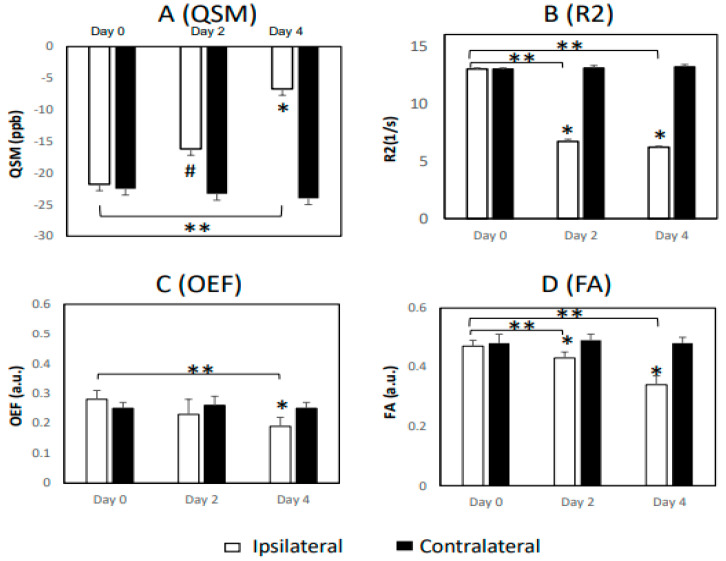
Demonstration of longitudinal changes in (**A**) magnetic susceptibility (QSM), (**B**) transverse relaxation rate (R_2_), (**C**) oxygen extraction fraction (OEF), and (**D**) fractional anisotropy (FA) in the white matter (WM) of ischemic monkey brains following stroke; # *p* = 0.08 and * *p* < 0.05, compared to the contralateral side; ** *p* < 0.05, compared to the days post-occlusion; ppb: parts per billion.

Ischemic infarction was seen in the cortical areas of the MCA territory of all monkeys. The infarction in one monkey brain (RJJ3) is illustrated on the diffusion-weighted image and overlapped on the corresponding T1-weighted images (baseline, pre-surgery) of the same monkey (Figure 1). No hemorrhage was observed in any stroke brain examined by SWI. The corresponding QSM, and R_2_, R_2_*, FA, MD maps of the monkey brain are also exhibited in Figure 1. Hemispheric comparisons of the MRI-derived values in gray matter and white matter are shown in Figure 2 and Figure 3, respectively. The results and details of the MRI parameter changes are also given in Table 1. 

The magnetic susceptibility (QSM) in gray matter was elevated significantly (17.5 ± 1.6 vs. 14.1 ± 0.6, or 24%, in ppb) during the hyperacute phase of stroke (day 0), and then it decreased substantially on day 2 (8.8 ± 3.1 vs. 14.7 ± 1.7, or −40%) and day 4 (8.4 ± 2.1 vs. 13.5 ± 1.8, or −38%) post-occlusion, as compared to that in the contralateral areas. Meanwhile, it was significantly reduced on day 2 and day 4 when compared to the day 0 result (*p* < 0.05; Figure 2), and no significant change in susceptibility was seen in the contralateral side (*p* > 0.1). Moreover, there was no significant change of susceptibility between day 2 and day 4. Significantly decreased R_2_ was seen on day 2 and day 4 in comparison with that in the contralateral side at each timepoint or that on day 0. MD decreased significantly on days 0, 2, and 4 when compared to that in the contralateral side, as well as temporally from day 0 to day 2, but no change was observed from day 0 to day 4 or from day 2 to day 4. The OEF in gray matter increased substantially (0.45 ± 0.08 vs. 0.36 ± 0.07, or 25%) during the hyperacute phase (day 0) and then decreased significantly on day 2 (0.28 ± 0.03 vs. 0.35 ± 0.09, or −20%) and day 4 (0.30 ± 0.06 vs. 0.38 ± 0.03, or −21%) when compared to the contralateral side at each timepoint. The OEF on day 2 and day 4 was decreased significantly in comparison with that on day 0. Moreover, there was no significant difference in OEF between day 2 and day 4. In addition, the temporal changes of OEF in grey matter were moderately correlated with MD (r = 0.52, *p* = 0.046) during acute stroke. 

A prior study indicated that the value of magnetic susceptibility of myelin is negative with water as a reference, and that severe demyelination can increase susceptibility up to zero [30]. As shown in Figure 3 for the results of white matter, no significant change in the susceptibility (QSM) was seen in white matter on day 0 (−21.8 ± 6.5 vs. −22.5 ± 4.5). However, it increased substantially on day 2 (−16.2 ± 4.3 vs. −23.3 ± 2.0, or 30%) and day 4 (−6.7 ± 2.2 vs. −24.0 ± 3.6, or 72%) post-occlusion, as compared to the contralateral areas of the brain (*p* < 0.05; Figure 3). Moreover, it increased progressively from day 0 to day 4, and substantial change was seen on day 4 in comparison to day 0. Similar to the findings in gray matter, a substantial decrease in R_2_ was observed in the lesion area compared to the contralateral area on day 2 and day 4 post-occlusion (*p* < 0.01), and it was substantially reduced when compared to that on day 0. In addition, a significant FA reduction in the ipsilateral regions of the white matter was seen on day 2 and day 4 when compared to the contralateral side, but no significant MD changes in white matter were seen at any timepoint post-stroke. No significant change of OEF in white matter was seen on day 0 or day 2, until day 4, when the OEF in white matter decreased substantially (0.19 ± 0.03 vs. 0.25 ± 0.02) post stroke. Additionally, no correlation was observed between the temporal changes in OEF and any DTI indices in white matter during acute stroke. Significant R_2_’ changes were seen in the white matter of the stroke brains from day 0 to day 4, but not in gray matter until day 4 (Table 1).

**Figure 4:**
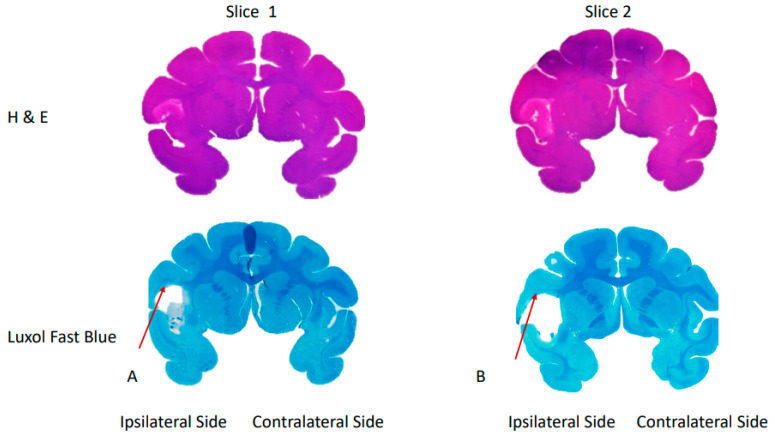
Illustration of representative slides of H&E staining (**top row**) and Luxol fast blue staining (**bottom row**) of a monkey brain (RRI3) at 96 h post-stroke. The arrows A and B mark the injured white matter fiber bundles in the stroke brain.

The H&E and Luxol fast blue staining results demonstrated substantial ischemic lesions in the infarct area and white matter of a stroke monkey brain (RRI3) harvested 96 h post-stroke, respectively (Figure 4). 

## 4. Discussion

In the present study, the longitudinal evolution of the oxygen extraction fraction (OEF) in the ischemic brain was revealed using a monkey model with permanent MCAO and QSM MRI techniques. Our results of OEF alteration in the gray matter of the brain during hyperacute (<7 h) and subacute (1–7 days) stroke were in good agreement with the previous findings in animals and patients [22,23]. It was also observed that the temporal changes in OEF were moderately correlated with those in the mean diffusivity of gray matter during acute stroke, suggesting that OEF alteration may be associated with cytotoxic edema during acute stroke. In contrast, OEF showed less sensitivity for characterizing white matter degeneration in the brain during acute stroke, demonstrating the substantial difference in the evolution of OEF between gray matter and white matter in the brain following stroke.

QSM changes in ischemic brains have been reported previously in studies of acute stroke animal models [7,8] and patients [31]. Compared to the contralateral hemisphere of the brain, an increase in magnetic susceptibility was seen in prominent cerebral vessels of injured regions in rodent brains with transient cerebral ischemia, and the regions of decreased susceptibility were observed at 24 h and 48 h after reperfusion, as reported previously [7]. In the subacute (1 day after stroke insult) phase of a canine model with pMCAO, increased susceptibility was detected in the infarct region of some animals in the study group with relatively high perfusion, while decreased susceptibility was found in the areas with decreased ADC and severe hypoperfusion in the other animals of the group [8]. As seen in the present study’s monkey model of stroke, the magnetic susceptibility in the lesion area was significantly higher than that in the contralateral area during the hyperacute phase, which could be explained by increased oxygen extraction leading to higher concentrations of deoxyhemoglobin in the veins, as reported in a previous QSM study of stroke rodents [7]. On day 2 and day 4 post-stroke, magnetic susceptibility in the lesion area was reduced substantially compared to the contralateral hemisphere of the brain, in agreement with a previous study in which the susceptibility value of cortical veins in the ischemic core was 38% lower than in the contralateral MCA territory in stroke patients scanned 24–72 h after successful thrombectomy [31]. 

Alteration of OEF during acute stroke has been investigated in previous studies of patients, as well as in rodent and baboon models. Elevated OEF indicates inadequate oxygen supply and was generally observed in the hyperacute phase (<7 h) of stroke in patients [11], as well as in transient MCAO studies of rat models [24] by QSM and baboon models by PET [23]. OEF reduction was generally seen in the subacute phase (1–7 days) of stroke, as reported previously in stroke baboons and patients [21,22,23]. The results of the present monkey model revealed the temporal evolutions of OEF in the gray matter of the brains from the hyperacute phase to the subacute phase of stroke, in good agreement with previous findings in animals and stroke patients [11,21,22]. Interestingly, the OEF’s temporal changes were consistent with the changes of susceptibility in the gray matter (Figure 2A,D) from day 0 to day 4, as well as in agreement with reduction of the transverse relaxation rates (R_2_) and MD during the subacute phase. In particular, OEF was correlated moderately with MD during acute stroke (Figure 2E), suggesting that changes in OEF may be associated with cytotoxic edema. Due to the small sample size and limited timepoints of the present study, further evaluation of the relationship between edema and OEF is warranted.

The temporal evolution of OEF in the brain following stroke was reported in a previous study of baboons with transient MCAO (tMCAO) and PET imaging [23]. OEF increased substantially during the initial 6 h occlusion and decreased at 1 h and 1–2 days after reperfusion in the baboons (compared to the contralateral hemisphere). Interestingly, the OEF was restored to the baseline after >15 days, and infarct lesions were ultimately not seen in any cortical regions of baboons but were identified in limited subcortical regions by MRI and H&E staining. The present work also demonstrated significant reductions of OEF in the cortical regions of monkeys from day 2 to day 4 post-stroke (Figure 2D). In contrast, the cortical tissues were irreversibly damaged in the monkey brain with permanent MCAO, suggesting that other measures such as perfusion MRI should be used with OEF to better assess the viability of ischemic brain tissue during acute stroke. 

Gray matter injury has been the main focus of investigation in stroke, as neuron loss in the motor cortex is closely associated with neurological disorders in stroke patients. Previous DTI studies have demonstrated that white matter is affected progressively following stroke insult, and the microstructural alteration is associated with prediction of stroke outcome [15,32]. White matter is vulnerable to ischemia, as oligodendrocytes are myelin-producing cells and highly sensitive to ischemia-induced oxidation, excitotoxicity, and inflammation [33], while loss of oligodendrocytes and subsequent myelin damage are the primary hallmarks of white matter lesions in stroke brains [34]. Compared to rodents with litter white matter, NHP brains are structurally and functionally similar to the human brain and have been suggested to be excellent stroke models in translational research on stroke [35,36,37]. 

The white matter degeneration in monkey brains following stroke has been examined previously using DTI, and the progressive evolution of microstructural integrity in gray matter and white matter was presented [38,39]. In particular, DTI showed excellent sensitivity to detect early alteration of white matter’s microstructure in the brain during hyperacute stroke, and obvious fiber damage was observed 48 h post-stroke in monkey brains with pMCAO [40]. As white matter has abundant myelin that is diamagnetic and its integrity affects the magnetic susceptibility of white matter, the QSM in white matter can be heavily affected by the integrity of the fiber bundles [30,41]. Increased susceptibility was observed in the white matter of mice at 5 h and 1 day following traumatic brain injury, and a decrease was seen thereafter [42]. 

The present study exhibited progressive changes in magnetic susceptibility in the white matter of stroke lesion regions during acute stroke. As shown in Figure 3A, the magnetic susceptibility values (~ −22 ppb) in white matter on the contralateral side of the stroke monkey brains were comparable to the results in previous reports of MS and PD patients measured at 3T [30,43]. Following stroke injury, the susceptibility in the stroke lesion area was not changed immediately during the hyperacute phase, but it substantially increased (the absolute values decreased) on day 2 and day 4, in agreement with the changes in the edema and demyelination process (reduction in R2 and FA, respectively; Figure 3B,D) of white matter at the same timepoints. In contrast, no significant reduction of OEF in white matter was seen until day 4 (Figure 3C). Obvious white matter damage was seen within 48 h post-stroke, as demonstrated in previous pathological results of the same cohort of monkeys [44]; the present findings indicate that QSM-derived OEF is less sensitive to characterize the degeneration of white matter bundles in the brain during acute stroke. 

R_2_’ is the difference between R_2_ and R_2_*, and it is related to oxygen extraction in the brain. It is assumed that R_2_’ is proportional to the product of OEF and relative cerebral blood volume (CBV) (R_2_’ = c × OEF × CBV, in which c is a tissue-specific constant) [45]. A significant reduction in R_2_’ was seen in the ischemic core area, and the increase in R2′ within hypoperfused regions of acute ischemic brains is most probably caused by the increase in local OEF, as indicated in a previous study of stroke patients [28]. R_2_’ mapping may be useful to differentiate the ischemic core from perfusion-impaired areas, as reported in a previous study of stroke patients [46]. Interestingly, the present study demonstrated a significant increase in R_2_’ in the injured white matter regions of stroke monkeys on day 0, but it was decreased from day 2 to day 4 (Table 1). In contrast, no significant change (decrease) in R_2_’ was seen in the injured gray matter regions until day 4 (Table 1). These findings may suggest that R_2_’ has better sensitivity to detect early changes in white matter following stroke insult than in gray matter. However, the temporal correlation of R2′ with OEF was not observed in either gray matter or white matter in the stroke monkeys, in disagreement with the findings from stroke patients [28], suggesting that the contribution of local CBV changes should be considered and further evaluated in future studies, as indicated in a previous patient study [45].

DWI-PWI mismatch has often been used to approximate the ischemic penumbra for the prediction of stroke infarct growth and assessment of reperfusion therapy benefits in stroke studies [17,47]. However, it can be complicated by benign oligemia, which is frequently seen in stroke patients and animal models [48]. In contrast, DWI-OEF mismatch was suggested to be more specific to define the ischemic penumbra compared to DWI-PWI mismatch, as reported in a study of stroke rodents [24]. The present findings in a monkey model of stroke demonstrate the effectiveness of QSM-derived OEF to assess the temporal changes in oxygen metabolism in the ischemic brain during hyperacute and subacute stroke using a clinical 3T scanner. In addition, the image quality of QSM-OEF could be further improved with ultrahigh-field (7T and above) MRI techniques, from which QSM benefits substantially [49,50]. Therefore, QSM-OEF has the potential to be a robust approach for the assessment of the viability of ischemic tissue and prediction of stroke outcomes with the increasing installation of 7T clinical scanners. However, further research is needed to confirm these findings and to determine how they might be translated into clinical practice.

## 5. Limitations

This study is limited in several respects. Frist, only one dataset was acquired during the <24 h after stroke onset. Therefore, the timepoints were limited to reveal the full dynamic evolution of OEF in the ischemic brain during the first 24 h. This is because prolonged and/or repeated exposure to isoflurane raises concerns for animal care when scanning nonhuman primates. Second, the T2*-weighted images were acquired using a 2D multi-echo GRE sequence, but not using a high-resolution 3D gradient-recalled echo sequence (with full flow compensation), which is generally used to acquire QSM data [4] due to the consideration of consistency in voxel sizes and dimensions between T2-, T2*-, and diffusion-weighted images. Third, the present study was conducted using rhesus monkeys with a permanent MCAO model. Fourth, QSM-derived OEF in gray matter has previously been validated with PET [19]. Estimation of OEF in white matter with QSM could be more complicated than that in gray matter, due to the tensor nature of magnetic susceptibility in white matter, which has abundant myelinated fiber bundles [51]. It was reported relative OEF is weakly affected by white matter orientation [52]. The mathematical relationship between the susceptibility and OEF in white matter should be investigated and validated in future studies. Finally, the sample sizes of the study (n = 8 on day 0, n = 6 on day 2, and n = 4 on day 4) were limited due to the high cost of using monkeys.

In conclusion, the preliminary results from our stroke model of nonhuman primates demonstrate that QSM-derived OEF is a robust approach to examine the progressive changes in gray matter in the ischemic brain from the hyperacute phase to the subacute phase of stroke. In addition, temporal alterations of oxygen metabolites might be associated with edema in the brain following the onset of stroke. Stroke is a leading cause of adult disability in the US, and it is associated with multiple risk factors, including hypertension, diabetes, obesity, and even COVID-19 infection [53]. The present findings from stroke monkeys suggest that QSM-derived OEF may provide complementary information to understand the neuropathology of stroke injury in the brain and could be useful for prediction of stroke outcomes.

## Figures and Tables

**Table 1:** Statistical results and *p*-values with Bonferroni correction (*: *p* <  0.05) by post hoc comparisons between hemispheres (ipsilateral (Ips.) vs. contralateral (Con.)) using a two-way multivariate analysis of variance (MANOVA), for magnetic susceptibility (QSM, ppb), R_2_ (1/s), R_2_’ (1/s), and OEF and diffusion metrics (FA, MD (10^−3^ mm^2^/s)) from white matter (WM) and/or gray matter (GM). The *p*-values less than 0.05 are presented in boldface type. Data are presented as the mean ± standard derivation. FA, fractional anisotropy; MD, mean diffusivity; OEF, oxygen extraction fraction.

Measurement	Day 0			Day 2			Day 4		
	Ips.	Con.	*p*	Ips.	Con.	*p*	Ips.	Con.	*p*
**WM, QSM**	−21.8 ± 6.5	−22.5 ± 4.5	0.91	−16.2 ± 4.3	−23.3 ± 2.0	**0.08**	−6.7 ± 2.2	−24.0 ± 3.6	**0.003** *
**WM, R_2_**	13.0 ± 0.1	13.0 ± 0.1	0.57	6.7 ± 0.2	13.1 ± 0.2	**<0.001** *	6.2 ± 0.1	13.2 ± 0.2	**<0.001** *
**WM, R_2_’**	12.1 ± 0.1	11.8 ± 0.1	**0.005** *	11.3 ± 0.2	11.8 ± 0.1	**0.05**	11.3 ± 0.1	11.8 ± 0.1	**0.001** *
**WM, OEF**	0.28 ± 0.03	0.25 ± 0.02	0.19	0.23 ± 0.05	0.26 ± 0.03	0.61	0.19 ± 0.03	0.25 ± 0.02	**0.02** *
**WM, FA**	0.47 ± 0.02	0.48 ± 0.03	0.47	0.43 ± 0.02	0.49 ± 0.02	**0.001** *	0.34 ± 0.03	0.48 ± 0.02	**0.003** *
**GM, QSM**	17.5 ± 1.6	14.1 ± 0.6	**0.04** *	8.8 ± 3.1	14.7 ± 1.7	0.13	8.4 ± 2.1	13.5 ± 1.8	**0.02** *
**GM, R_2_**	10.2 ± 0.2	10.4 ± 0.1	0.19	7.0 ± 0.1	10.5 ± 0.1	**<0.001** *	6.8 ± 0.3	10.4 ± 0.2	**<0.001**
**GM, R_2_’**	9.9 ± 0.3	9.7 ± 0.2	0.19	9.6 ± 0.2	9.6 ± 0.2	0.99	9.1 ± 0.2	9.6 ± 0.1	**0.04** *
**GM, OEF**	0.45 ± 0.08	0.36 ± 0.07	0.05	0.28 ± 0.03	0.35 ± 0.09	**0.01** *	0.30 ± 0.06	0.38 ± 0.03	**0.04** *
**GM, MD**	0.75 ± 0.03	0.84 ± 0.02	**0.002** *	0.66 ± 0.06	0.84 ± 0.02	**0.001** *	0.70 ± 0.05	0.85 ± 0.02	**0.02** *

## Data Availability

Data for this study have not been publicly archived and are available upon reasonable request from the corresponding author.
